# Species *Fowl aviadenovirus B* Consists of a Single Serotype despite Genetic Distance of FAdV-5 Isolates

**DOI:** 10.3390/v14020248

**Published:** 2022-01-27

**Authors:** Győző L. Kaján, Anna Schachner, Ákos Gellért, Michael Hess

**Affiliations:** 1 Eötvös Loránd Research Network, Veterinary Medical Research Institute, 1143 Budapest, Hungary; gellert.akos@vmri.hu; 2 Christian Doppler Laboratory for Innovative Poultry Vaccines (IPOV), University of Veterinary Medicine, 1210 Vienna, Austria; anna.schachner@vetmeduni.ac.at; 3 Clinic for Poultry and Fish Medicine, Department for Farm Animals and Veterinary Public Health, University of Veterinary Medicine, 1210 Vienna, Austria; michael.hess@vetmeduni.ac.at

**Keywords:** fowl adenovirus, molecular typing, fowl adenovirus 5, serotyping

## Abstract

Fowl adenoviruses (FAdVs) are infectious agents, mainly of chickens, which cause economic losses to the poultry industry. Only a single serotype, namely FAdV-5, constitutes the species *Fowl aviadenovirus B* (FAdV-B); however, recently, phylogenetic analyses have identified divergent strains of the species, implicating a more complex scenario and possibly a novel serotype. Therefore, field isolates of the species were collected to investigate the contemporary diversification within FAdV-B, including traditional serotyping. Full genomes of fourteen FAdV-B strains were sequenced and four strains, possessing discriminatory mutations in the antigenic domains, were compared using virus cross-neutralization. Essentially, strains with identical antigenic signatures to that of the first described divergent strain were found in the complete new dataset. While chicken antiserum against FAdV-5 reference strain 340 could not neutralize any of the newly isolated viruses, low homologous/heterologous titer ratios were measured reciprocally. Although they argue against a new serotype, our results indicate the emergence of escape variants in FAdV-B. Charge-influencing amino acid substitutions accounted for only a few mutations between the strains; still, these enabled one-way cross-neutralization only. These findings underline the continued merit of the cross-neutralization test as the gold standard for serotyping, complementary to advancing sequence data, and provide a snapshot of the actual diversity and evolution of species FAdV-B.

## 1. Introduction

Adenoviruses possess an icosahedral, non-enveloped capsid and a linear, non-segmented, double-stranded DNA genome, and infect all classes of vertebrates [1,2]. The family *Adenoviridae* was recently included in the realm *Varidnaviria*, kingdom *Bamfordvirae*, phylum *Preplasmiviricota*, class *Tectiliviricetes* and order *Rowavirales* [3]. Within the family, the members of the genus *Aviadenovirus* infect exclusively birds [4]. Strains of the species *Fowl aviadenovirus A*–*E* can cause severe economic losses for the poultry industry, especially in broiler production [5,6]. These five species are further divided into 12 serotypes (FAdV-1-8a and 8b-11), and there is a single serotype, fowl adenovirus 5 (FAdV-5), classified into the species *Fowl aviadenovirus B* (FAdV-B) [7,8].

Available clinical data regarding this species and its sole serotype are relatively limited compared to other FAdV species. All other FAdV species contain certain serotypes which are the etiological agents of well-characterized chicken diseases (inclusion body hepatitis, hepatitis–hydropericardium syndrome and gizzard erosion) [9]. In contrast, FAdV-B strains were detected from various pathologies (arthritis, enteritis, inclusion body hepatitis and hemorrhages, among others), but no primary disease was associated with them [10,11,12,13]. The FAdV-5 prototype strain 340 was isolated decades ago during a routine survey carried out in hens without specific pathology [7].

Our recent results demarcated a proposed new genotype within the species: strain 40440-M/2015 Debrecen (hereafter strain 40440) shared lower sequence identity with reference strain 340, in the relevant antigenic regions, than the closest related known FAdV serotypes shared with each other [12]. Thus, we wanted to investigate if this newly recognized level of genetic diversity in FAdV-B has consequences on serum neutralization, and to scrutinize the possibility of a novel serotype within the species. Furthermore, our results shed new light on the actual diversity of FAdV-B, whose molecular evolution has remained with many gaps due to the lack of priority given to this fowl aviadenovirus species in regard to clinical manifestations.

## 2. Materials and Methods

### 2.1. FAdV-B Isolates

All FAdV-B strains included in this study were isolated from commercial chickens from different European countries during the years 2015–2018. The origin of the strains, along with the clinical documentation for the corresponding flock, if available, are summarized in Table 1.

The complete genome of strain 40440 (MG953201) has been reported previously [12]. The remaining fourteen strains were obtained from samples submitted for routine diagnostics to the Clinic for Poultry and Fish Medicine, University of Veterinary Medicine, Vienna, Austria. The presence of FAdV-B strains in these samples was determined, after propagation on chicken embryo liver (CEL) cells, by amplification and sequencing of the hexon loop-1 (L1) gene region [14]. Prior to further processing, all isolates were plaque purified 3 times. For this, 0.1 mL of serial tenfold virus dilutions were inoculated on a CEL monolayer grown in 6-well plates, which were afterwards incubated with 3 mL tissue culture medium. After 24 h, the medium was replaced by 3 mL M199 tissue culture medium containing 1.26% agarose 4% (Thermo Fisher, Vienna, Austria). Upon visible formation of plaques, individual plaques were excised from the agarose layer using a 1000 μL pipette tip with an orifice cut with a sterile scissor to fit the plaque. The plaque was transferred to an Eppendorf tube and dissolved in 1–2 mL medium, to serve as inoculum for successive rounds of plaque purification.

### 2.2. Sequencing and Genome Assembly

Whole-genome sequencing of the 14 strains was performed on an Illumina platform (MiSeq V3, Central Service Facility NGS Unit, Vienna, Austria). Paired-end libraries were generated, and samples were multiplexed in a single lane, separated by barcoding. Contaminating chicken genome reads were excluded by mapping them against the *Gallus gallus* genome (NCBI Genome GRCg6a, RefSeq assembly GCF_000002315.6) using MIRA 4.0.2 [15]. The resulting reads were error corrected and normalized to a 40-times coverage using BBNorm from the BBTools suite. The normalized reads were quality trimmed, and then mapped to the genome of reference strain 340 (KC493646) using the Geneious mapper at the highest sensitivity, and allowing it to reveal structural variants, insertions and deletions of any size [16]. For the resulting consensus sequences, the same reads were mapped using the same sensitivity settings to obtain the final consensus sequences. Genome annotations were transferred from strain 40440 using Geneious and manually checked and edited. The exported GenBank flat files were converted to five-column, tab-delimited feature tables using the GB2sequin for submission purposes [17]. The complete genome sequences were deposited in the GenBank, under accession numbers OK283042-OK283055, whereas sequence reads were archived under the NCBI BioProject, under the accession number PRJNA781911.

### 2.3. Phylogenetic Analysis

Phylogeny was inferred using pairwise sequence identity analyses and phylogenetic tree reconstructions.

Pairwise sequence identities were measured using MAFFT for alignments in the Sequence Demarcation Tool [18]. Sequence identities were calculated based on the complete genomes and on the amino acid (aa) sequences of: the complete DNA polymerase, penton base, hexon and fiber, the hexon hypervariable loop-1, and the fiber knob, as well as the concatenate of the major capsid proteins’ serodeterminant regions (penton base, hexon loop-1, hexon loop-2 and fiber knob).

**Table 1:** FAdV-B isolates investigated in this study.

Strain Name (GenBank acc.no.)	Country, Year of Origin	Type of Sample	Clinical Signs, Pathologies	Other Bacterial/Viral Findings
14-24408 (OK283042)	Austria ^a^, 2014	Organ pool (liver, intestine, caecal tonsils)	Birds dead on farm	Viral: none
15-368 (OK283043)	Austria ^b^, 2015	Organ pool (liver, intestine, caecal tonsils)	Broilers, dead on farm	Viral: none
15-1401 (OK283044)	Austria ^c^, 2015	Organ pool (liver, intestine, caecal tonsils)	Birds dead on farm	Viral: none
15-3466 (OK283045)	Austria ^d^, 2015	Organ pool (liver, intestine, caecal tonsils)	Broilers, dead on farm No lesions recorded	FAdV-1
15-4225 (OK283051)	France, 2015	Pancreas	21-day-old chickens with severe tenosynovitis (2–3% incidence)	Tendons: Reovirus
15-4616 (OK283046)	Austria ^e^, 2015	Organ pool (liver, intestine, caecal tonsils)	Broilers, dead on farm Liver swollen, perihepatitis Pericarditis Ascites	*E. coli*, *Staphylococcus* spp. Reovirus
15-6270 (OK283047)	Austria ^f^, 2015	Organ pool (liver, intestine, caecal tonsils)	Broilers, dead on farm Liver swollen, necroses Ascites Intestinal dilatation, foamy contents	*Proteus* spp. in all organs
15-6541 (OK283048)	Austria ^g^, 2015	Organ pool (liver, intestine, caecal tonsils)	Broilers, dead on farm	*E. coli*, *Staphylococcus* spp.
17-25702 (OK283049)	Hungary ^a^, 2017	Caecal tonsils	Broiler breeders, dead on farm	Viral: none
18-907 (OK283050)	Hungary ^b^, 2018	Caecal tonsils	Broiler breeders, dead on farm	Viral: none
18-6238 (OK283052)	Germany, 2018	Cell culture supernatant (material of origin unknown)	n.r.	n.c.
18-6239 (OK283053)				
18-11753 (OK283054)	Hungary ^a^, 2018	Caecal tonsils	Broiler breeders, dead on farm	n.c.
19-7207 (OK283055)	Hungary ^c^, 2018	Cloacal swab pool	n.r.	n.c.

^a–g^ Samples from the same country, but separate farms in different locations, are indicated by different superscript letters. n.r.—not reported; n.c.—not conducted.

The strains were compared phylogenetically to FAdV-5 reference strain 340 based on tree inferences on the complete genomes and complete hexon aa sequences. Furthermore, a phylogenetic tree was inferred using all FAdV-B (NCBI: txid190062) hexon loop-1 nucleotide sequences. Here, misannotated and low-quality sequences were excluded from the analysis after a preliminary affirmatory molecular typing and quality check. Misannotation is, unfortunately, not limited to FAdV-B sequence entries; therefore, the strain 340 hexon loop-1 nucleotide sequence was used as a query in an NCBI BLASTn search to find further virus strains not listed in NCBI Taxonomy ID 190062 (species FAdV-B). As entry MK757473 (erroneously designated as fowl aviadenovirus 5 isolate LYG, BLASTX score: 494) was found to not represent a FAdV-B strain, a BLASTX score threshold >494 was applied. Analyzed strains are summarized in Table 2. Sequence alignments were conducted using MAFFT: the E-INS-i algorithm was used for the genomes and the G-INS-i for both hexon alignments [19]. The alignments were edited using Gblocks. Evolutionary models were predicted using ModelTest-NG: the GTR + I + G model was selected for the genomes and the partial hexons, whereas the JTT + I + G was selected for the complete hexons [20]. Phylogenetic trees were reconstructed using RAxML-NG; the robustness of the trees was tested with a non-parametric bootstrap calculation using 1000 repeats. Phylogenetic trees were visualized using MEGA 7 [21], and bootstrap values were given as percentages if they reached ≥75%.

### 2.4. Cross-Neutralization Testing

Cross-neutralization testing was performed in order to examine the serological relationships among the viruses, as well as to assess the possible effects of sequence differences on mutual recognition between current field isolates of this study and strain 340, the FAdV-5 (FAdV-B) reference strain, isolated in Northern Ireland around 1970 [7]. Field strains included were 40440 and three representatives (15-6541, 15-4225, and 18-6238) from the remaining FAdV-B strains. The latter three were selected based on the complete genome phylogenetic analysis to represent relatively distant strains; however, all three selected strains shared complete aa sequence identity in the major capsid proteins among each other, while featuring few aa differences compared to 40440 and 340.

Monospecific antisera were raised against each strain in specific pathogen-free chickens (VALO BioMedica GmbH, Osterholz-Scharmbeck, Germany) immunized intramuscularly with a mixture of inactivated (1% formaldehyde) virus and adjuvant (GERBU LQ no. 3000, GERBU Biotechnik GmbH, Heidelberg, Germany). The procedures used on the experimental birds were discussed and approved by the institutional ethics committee and the national authority according to §26 of the Law for Animal Experiments, Tierversuchsgesetz 2012–TVG 2012 (license numbers GZ 68.205/0158-WF/V/3b/2014, GZ 68.205/0044-WF/V/3b/2016).

Neutralizing antibody (nAb) titers of the freshly prepared sera were pre-determined in a microtiter assay on CEL cells, testing each serum in duplicate against its corresponding (homologous) virus. Heat inactivated, serially diluted (log_2_3–log_2_14) sera were incubated with 100 TCID_50_ of the virus. After five days of incubation at 37 °C in 5% CO_2_, the wells were checked for cytopathic effect (CPE) using light microscopy. For the subsequent cross-neutralization assay, the sera were standardized to a concentration of 20 serum units against the homologous strain, diluted at a ratio of 1:20, then tested in serial two-fold dilutions against all viruses under the conditions outlined above [22].

An 8-fold difference in the titer compared to the homologous value was assumed for serotype differentiation [7,23].

**Table 2:** *Fowl aviadenovirus B* strains, phylogenetically analyzed based on their hexon loop-1 nucleotide sequences. The accession numbers of the newly sequenced strains are given in bold.

Accession Number	Strain Identifier	Country of Origin	Year of Origin	Taxonomic Lineage (If not FAdV-B)
AF508953	TR22	Japan	1960s	
KC493646	340	Northern Ireland	1970s	
EF442425	Rostov/2007/02/chicken/B	Russia	2007	*Aviadenovirus*
FN869989	08-21472	Austria	2008	
FN869990	08-8669	Austria	2008	
FN869987	09-7470-2	Hungary	2009	
FN869988	09-7473-2	Hungary	2009	
FN869991	09-6893	Hungary	2009	
HQ697592	K318/09	South Korea	2009	*Fowl aviadenovirus D*
JF304111	8844	Hungary	2010	
KC750798	160	Hungary	2011	
KC750799	177	Hungary	2011	
KP274034	FAdVB_CGOU224	Cote d’Ivoire	2012	unclassified aviadenovirus
KP274035	FAdVB_CPON047	Cote d’Ivoire	2012	unclassified aviadenovirus
KP274036	FAdVB_CPAU286	Cote d’Ivoire	2012	unclassified aviadenovirus
KP274037	FAdVB_CPON040	Cote d’Ivoire	2012	unclassified aviadenovirus
KP274038	FAdVE_CDAO182	Cote d’Ivoire	2012	unclassified aviadenovirus
KP828383	GB 1643	Germany	2012	
MG953228	9892	Hungary	2013	
MT500572	D2453	Ukraine	2013	
MG953211	2255	Hungary	2014	
**OK283042**	14/24408	Austria	2014	
MG953201	40440	Hungary	2015	
MG953219	45871	Hungary	2015	
MG953222	5626	Hungary	2015	
MG953223	70147	Hungary	2015	
**OK283043**	15/368	Austria	2015	
**OK283044**	15/1401	Austria	2015	
**OK283045**	15/3466	Austria	2015	
**OK283046**	15/4616	Austria	2015	
**OK283047**	15/6270	Austria	2015	
**OK283048**	15/6541	Austria	2015	
**OK283051**	15/4225	France	2015	
MK509019	NGR_FAdV_Ch2	Nigeria	2017	*Fowl aviadenovirus C*
**OK283049**	17/25702	Hungary	2017	
**OK283050**	18/907	Hungary	2018	
**OK283052**	18/6238	Germany	2018	
**OK283053**	18/6239	Germany	2018	
**OK283054**	18/11753	Hungary	2018	
**OK283055**	19/7209	Hungary	2019	
AF339916	ATCC VR-828, VR-1854, IBH-2A	n.a.	n.a.	*Fowl aviadenovirus D*
AF508953	TR22	Japan	1960s	
KC493646	340	Northern Ireland	1970s	
EF442425	Rostov/2007/02/chicken/B	Russia	2007	*Aviadenovirus*
FN869989	08-21472	Austria	2008	
FN869990	08-8669	Austria	2008	

n.a.—not available.

### 2.5. Modelling of the Major Capsid Proteins

In spite of a notable genetic distance, in other words, a relatively low sequence identity, the divergent FAdV-B strains did not represent a novel serotype. Therefore, to determine the structural background of this, the three-dimensional protein models of the serodeterminant major capsid proteins were generated for the reference (340) and the divergent FAdV-5 strain (40440), as well as an FAdV-8a strain (TR59), based on the following aa sequences: AGL34683, QCC26485 and ANJ02525 (penton base); AGL34687, QCC26479 and ANJ02529 (hexon); and finally AGL34695, QCC26484 and ANJ02537 (fiber). FAdV-8a served as a positive control, as it represented a clearly distinct serotype compared to any FAdV-5 strain. All models were generated using the Robetta structure prediction server [24]. Not only did this application generate the monomer models, but it also created the appropriate biological assemblies: pentamer from penton base, and trimers from hexon and fiber subunits. The output protein model structures were refined with the MacroModel energy minimization module of the Schrödinger Suite [25] to eliminate steric conflicts between the side-chain atoms. The models were visualized in VMD version 1.9.3 [26]. Electrostatic potential maps were calculated with Adaptive Poisson–Boltzmann Solver (APBS) version 1.3 using the linearized Poisson–Boltzmann method [27] with a dielectric constant of 78.0 and 2 for the water solvent and the protein core, respectively. The partial charges in the electrostatic potential were calculated with PDB2PQR [27].

## 3. Results

### 3.1. Propagation of FAdV-B Strains

Five days after the infection of CEL cells, every FAdV-B strain developed a visible CPE including plaque formation, rounding and condensation of cells, and detachment from the plate, similar to those caused by the reference strain 340. No major differences were recorded in the morphology and/or the progression of CPE between investigated strains.

Five strains’ infective titers were determined in the course of cross-neutralization testing (see Section 3.4); these were 10^4.4^ TCID_50_/mL for strain 340, 10^6.7^ TCID_50_/mL for strain 40440 and 18-6238, 10^8.2^ TCID_50_/mL for strain 15-4225, and 10^9^ TCID_50_/mL for strain 15-6541.

### 3.2. Genome Sequences

The complete genome sequences of the 14 FAdV-B strains were determined. The genome lengths varied between 45.741 and 45.838 bp, with a G + C content of 56.5–56.6%. After the final sequence read mapping, the final read coverage minimums were between 5 and 11, and the means were between 76.4 and 80.3. The same open reading frame content and orientation of strain 40440 was observed in every new FAdV-B genome with 36 open reading frames, inverted terminal repeats of 86 bp, and two tandem repeat regions; the first one was located in the intergenic regions between ORF27 and GAM-1, and the second between GAM-1 and ORF17.

### 3.3. Phylogenetic Analysis

**Figure 1:**
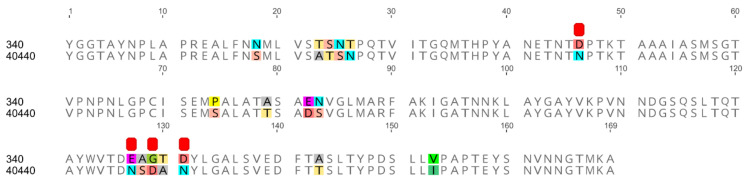
Amino acid alignment of hexon loop-1 sequences originating from two fowl adenovirus 5 strains: the reference strain 340 and another one (40440). The newly sequenced divergent strains are identical on the amino acid level to strain 40440 on this stretch. Differences are highlighted; furthermore, amino acid substitutions affecting the electrostatic charge of the protein are denoted using red squares.

All serodeterminant surface antigens shared 100% aa sequence identity among the newly sequenced strains; the penton base and the hexon genes shared 100% nucleic acid identity too, whereas the fiber genes had 0–2 nucleotides in difference. Additionally, the penton base, hexon and fiber knob of strain 40440 shared 100% aa identity with the analyzed strains’ corresponding proteins. The same proteins shared a lower sequence identity (89.80–99.63%) with those of strain 340. In the DNA polymerase aa sequences of the newly sequenced strains, 0–2 differences were counted. The sequence identity values are summarized in Table 3, and the alignment of the hexon loop-1 is available in Figure 1.

**Figure 2:**
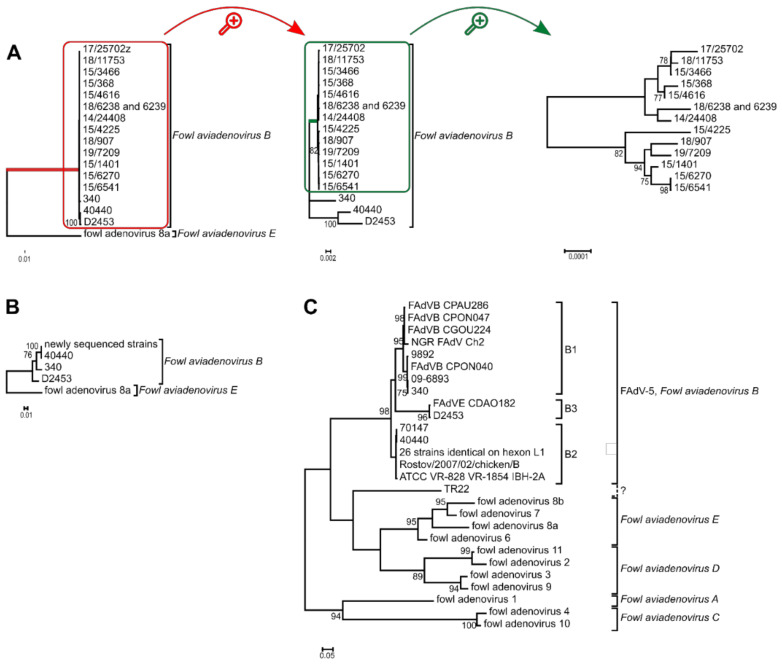
Phylogenetic analysis of *Fowl aviadenovirus B* strains based on the (**A**) complete genome sequence, (**B**) the hexon amino acid sequence and the (**C**) hexon loop-1 (L1) nucleotide sequence. Trees (**A**,**B**) were rooted on fowl adenovirus 8a, whereas tree (**C**) was midpoint rooted. (**A**) Branch lengths were short within the species *Fowl aviadenovirus B*; thus, the red highlighted branch of the species is shown separately. From this tree, the green highlighted branch of the newly sequenced strains was separated again to reveal branch lengths among these strains too. (**C**) The 26 identical strains: 160, 177, 2255, 5626, 8844, 45871, 08-21472, 08-8669, 09-7470-2, 09-7473-2, 14/24408, 15/1401, 15/3466, 15/368, 15/4616, 15/6270, 15/6541, 17/25702, 18/11753, 15/4225, 18/6238, 18/6239, 18/907, 19/7209, GB 1643, K318/09. See Table 2 for further details.

Based on the complete genomes’ phylogenetic tree reconstruction, all newly sequenced strains were placed on a monophyletic branch, and strain 40440 was most closely related to the recently described recombinant D2453 [13]. Analyzing the hexon aa sequences, it was confirmed again that the newly analyzed FAdV-B strains share an identical hexon protein with strain 40440. Furthermore, the analysis of the hexon loop-1 nucleotide sequences clustered almost all FAdV-B strains into three clades, members of which shared almost identical loop-1 nucleotide sequences. Only strain TR22 constituted an independent branch, that was not monophyletic with other FAdV-B members; furthermore the distance of TR22 from other FAdV-B strains was similar to the observable interspecies distances. The phylogenetic tree reconstructions are shown in Figure 2.

### 3.4. Neutralizing Antibodies and Cross-Neutralization of FAdV-B Strains

Neutralizing titers, determined in sera collected at 28, 35 and 42 days post immunization, were in the upper range of the routinely tested interval for FAdV-5 reference strain 340 (log_2_11–log_2_12), and field strain 18-6238 (log_2_12–log_2_13). Since no end point could be determined within this range for any of the remaining antisera, additional doubling serum dilutions up to log_2_20 were included. This revealed titers of log_2_14–log_2_15 for 40440 antisera, while injections with strains 15-4225 and 15-6541 revealed antisera reaching log_2_16–log_2_18 and log_2_18–log_2_19, respectively. A small but consecutive rise in titer between the first and last time point was recorded in each tested individual, indicating that nAb production was ongoing 4–6 weeks after the antigen injection.

In the cross-neutralization assay, mutual recognition occurred between all contemporary FAdV-B field strains, including the genetically most distant 40440 (Table 4). An identical level of neutralization was shown between the three newly sequenced field strains, and an up to two-fold lower neutralization compared to the respective homologous titer of 40440. Antisera against all four current field strains were also able to neutralize reference virus 340, with a two- to four-fold titer reduction compared to the homologous reaction. However, all of the reactions with 340 were unanimously one-directional, as the antiserum against 340 exhibited no neutralizing activity against any of the current FAdV-B field isolates.

### 3.5. Modelling of the Major Capsid Proteins

**Figure 3:**
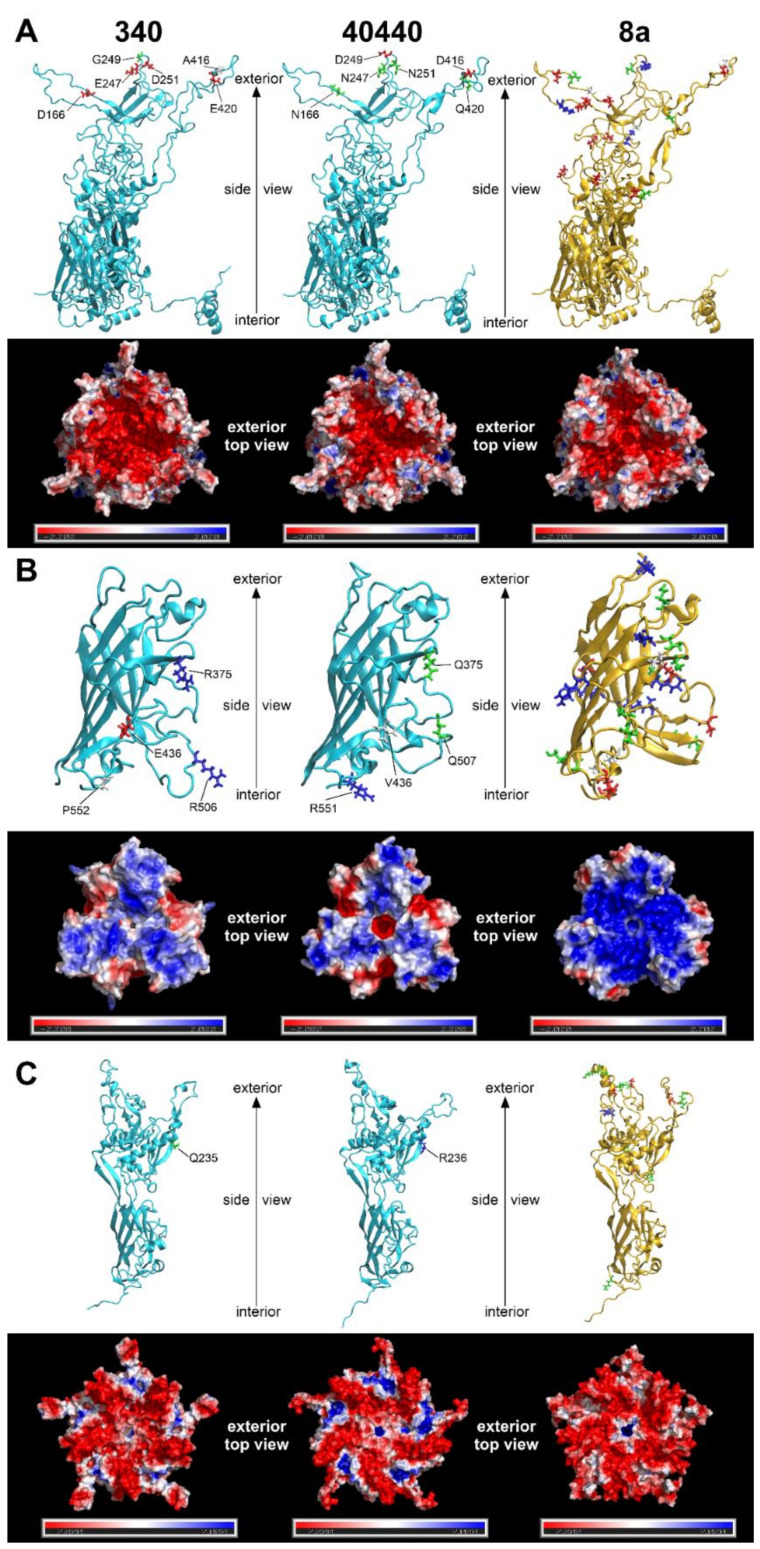
Three-dimensional major capsid protein models of strains 340 and 40440 (both serotype fowl adenovirus 5 [FAdV-5]) and FAdV-8a: (**A**) complete hexon; (**B**) fiber knob (amino acid stretches for strain 340: 349–553, 40440: 349–554, FAdV-8a: 314–524); and (**C**) penton base (amino acid stretches for strain 340: 76–542, 40440: 77–543, FAdV-8a: 81–552). The upper strips of each subset show the monomer subunits in cartoon representation; here, amino acid substitutions affecting the electrostatic charge of the protein are highlighted. For strains 340 and 40440, substitutions compared to each other, and for fowl adenovirus 8a, substitutions compared to strain 340, are highlighted. Red and blue are charged (negatively and positively, respectively), green are polar, and white are nonpolar amino acids. The lower strips of each subset represent the electrostatic surface potentials of the trimeric (hexon and fiber knob) or pentameric (penton base) proteins, where red color represents regions with a potential value below −2.0 kT, white represents 0.0 kT, and blue represents regions above +2.0 kT.

The predicted structures of capsid proteins are presented in Figure 3. On the hexon trimers, strain 40440 had a positively charged surface potential patch in its peripheral region; this patch was smaller and less positively charged in FAdV-8a, and was neutral in strain 340. The central, negatively charged depression was most extensive in strain 340, whereas it was similar in size in strain 40440 and FAdV-8a. On the fiber trimers, a negatively charged peripheral indentation was present in both FAdV-5 strains, though it was more prominent in strain 40440. In the latter one, a small, marked negatively charged region was observed around the central cavity, too, whereas the FAdV-8a fiber head was predominantly strongly positively charged almost on its entire surface. On the penton pentamer, positively charged dents were observed in FAdV-5 strains, which were charged more strongly in strain 40440. The dents were observable in FAdV-8a, too, but were uncharged. A positively charged small central cavity was observed both in strain 40440 and in FAdV-8a; the surrounding region was slightly negative in strain 40440 and more neutral in FAdV-8a.

Differences in the total quantity of charged aas were also compared between strains 340 and 40440 based on Figure 3. On a hexon monomer, four negatively charged aas were counted on strain 340, and two on strain 40440. On a fiber monomer, two positively charged and a negatively charged aa were counted on strain 340, and a single positively charged aa was noted on strain 40440. Finally, on a penton base monomer, a single positive aa was noted on strain 40440. This analysis compared only the total quantity of the charged aas, but all aa changes were scrutinized, focusing on charge alterations based on sequence alignments. These amino acid changes of the major capsid proteins are summarized in Table 5.

## 4. Discussion

In previous studies, we had proposed the demarcation of a second genotype for genetically divergent strains within species FAdV-B [10,11], based on the finding that the genetic distances between the divergent and the official FAdV-5 reference strains had been higher on every analyzed genomic stretch than those measured between the closest related, acknowledged serotype [12]. These results included all adenoviral serodeterminant surface antigens and a concatenate of these; thus, it was assumed that such a genetic distance predicts a novel serotype, demarcated by serum neutralization. By definition, a serotype either exhibits no cross-reaction with others, or shows a homologous/heterologous titer ratio of greater than 8 (in both directions) [7,23]. Four contemporary divergent FAdV-B strains—sharing identical sequences in the major adenoviral serodeterminant regions—were tested in this study, together with reference strain 340 in a virus cross-neutralization assay. In this setting the historical prototype 340 was singularized from the group of contemporary strains by the fact that its respective antiserum completely failed to neutralize any of the contemporary strains; these, less surprisingly, shared full recognition among each other underlining their close serological relationship. On the other hand, the homologous/heterologous titer ratios of antisera against the divergent strains were below the minimal threshold value of 8; this, by definition, contests a presumptive new serotype within species FAdV-B.

The major capsid proteins of adenoviruses are the hexon, the penton base and the fiber [2], and the external surface of these, mainly the hypervariable loops of the hexon protein and the fiber knob [28,29], act as antigenic determinants [23,30,31,32]. Genetic changes in these surface proteins are a predictor for serological distancing [10,28]; thus, the major capsid proteins were modelled to elucidate why the serodeterminants—despite genetic distance—did not result in a different neutralization phenotype in the actual case. Due to its numerical abundance in the capsid [33], the hexon loop-1 is the dominant component of the serotyping response [34,35]. Although 26 aas were substituted in the divergent strains’ complete hexon compared to that of strain 340 (Figure 1), none of these reversed the charge of the residue in question, and only one caused a nonpolar-to-charged aa change. A further five substitutions altered the charge of the residue only slightly by a change from a polar acidic aa to a still-polar but uncharged one, or vice versa. Similarly, charge reversal was not observed in any residue of the fiber knob or the penton base; only nonpolar-to-charged or polar-to-charged changes were observed. Conversely, in a clearly distinct serotype, serotype FAdV-8a, a higher number of marked charge alterations (Table 5) and an apparently different fiber knob electrostatic surface potential (Figure 3) were observed. After analyzing the substitutions and the resulting electrostatic surface potentials, we conclude that genetic distance alone might be insufficient as a criterion for serotype inference of fowl adenoviruses. Consequently, to acquire a more detailed understanding of the molecular basis for neutralization phenotypes, comparative bioinformatics approaches—taking into account the location of changed residues within the molecules´ tertiary structure and corresponding changes of the electrostatic surface potentials—should also be included, alongside sequence divergence in future studies.

The unidirectional recognition between the strains indicates (Table 4), however, that the similarity of all serodeterminant components was, in summary, still enough to enable at least a one-way cross-neutralization. On the other hand, the observed amino acid changes in the capsid proteins (Figure 1 and Figure 2B, Table 3 and Table 5) cause the divergent strains to be on the borderline of representing a new serotype. Although not a new serotype, all divergent strains, according to their neutralization behavior, represent prime strains of FAdV-B. A prime strain induces antibodies that neutralize the originally recognized prototype strain, while it is, in turn, poorly (or not) neutralized by the prototype antiserum. This phenomenon has been described already among species FAdV-D and -E strains [35]. To investigate the possibility that only one of the components, and its cognate antibody fraction, was responsible for heterologous recognition, and that a lack thereof caused one-sided failure to neutralize (i.e., antiserum to strain 340 vs. the divergent viruses), we screened the antibody contents of all of the tested sera using Western blots with whole virus preparations. Although Western blotting might also detect antibodies against non-neutralizing epitopes the blots still indicated the presence of the full capsid-directed antibody repertoire in all of the sera (data not shown). This makes it less likely that the antiserum to strain 340 was devoid of a specific antibody fraction, and that this is an explanation for accidentally-assigned prime strains. Amino acid differences in the hexon might well contribute to the noticed one-way neutralization (Figure 1). In addition, the complete lack of neutralization of the closely related strains by serum raised against reference strain 340 argues for additional mechanisms. Thus, we turned, again, to the conformational and surface potential models.

One of the conditions required for basic protein–protein interactions is electrostatic complementarity. In our case, the hexon of strain 340 carried exactly twice as many negative charges (Figure 3A) in the antigenic L1 and L2 regions as strain 40440, which is the prime strain. Thus, antibodies, raised against the reference strain’s hexon have twice as many positive charges as those against the prime strain hexon. Simultaneously, the hexon of the prime strain bears significant positively charged surface regions that are likely to repel the more positive antibodies raised against the reference strain, so no neutralization occurs. Conversely, antibodies raised against the hexon of a prime strain with half the number of positive charges are capable of binding to the less positively charged hexon of the reference strain, leading to successful neutralization. The exact description of cross-neutralization is further complicated by the fact that, in addition to the hexon, two other proteins—the fiber knob and penton base—also have antigenic regions. In the present case, however, the antigenic regions of the fiber knob and penton base of the reference strain are similar to those of the prime strain, so these proteins probably do not contribute to this prime strain phenomenon. Finally, it needs to be kept in mind that antibodies against different capsid proteins have synergistic potential with regard to neutralizing activity [36].

Essentially identical antigenic sets with the serodeterminants of 40440 were found in all of the newly sequenced viruses, though they spanned a period of four years and a sampling region of four countries. Although other variants had been detected earlier [10,11,37], this serodeterminant set was so prevalent in our study that we speculate that it represents the currently dominating wild type having replaced the original prototype. Unfortunately, FAdV-B is by far the most underrepresented of all of the species, with regard to its documentation throughout the decades, from the isolation of strain 340 to recent strains; this is mainly due to a lack of clinical relevance attributed to those strains. However, the finding that even the highly conserved, slowly evolving DNA polymerase gene showed greater variability among the strains than the surface antigens suggests that this serodeterminant set was acquired long before its recent detection [38,39]. Furthermore, the analysis of all of the available FAdV-B hexon loop-1 sequences (Figure 2C) revealed that 75% of the strains clustered into this genotype, genotype B2 [10]. It is tempting to hypothesize that this antigenic set might give an evolutionary advantage to the strains, and we can observe a shift in the serodeterminants of FAdV-5. The new strains were already not neutralized by the antiserum of the reference strain; on the other hand, any sera against the new, divergent strains (genotype B2) neutralized the reference strain (genotype B1). This alone already provides an evolutionary advantage for the divergent strains; these strains are possibly capable of infecting chickens already seroconverted against the reference strain, whereas the opposite is unlikely true with the presence of neutralizing antibodies. Such a hypothesis of antigenic shift should be handled cautiously, as the average life expectancy of a broiler chicken is only 5–7 weeks. Although not impossible, it is not probable that two adenovirus infections will occur during this short period; thus there would be no evolutionary pressure for such a shift. On the other hand, breeders or layer pullets live much longer, so there is a theoretical possibility for such an explanation. Concurrent infections, with more than one adenovirus type, do occur in chickens just as in humans [11,14,40,41,42,43,44,45,46,47]; this is now strengthened in our own dataset by the example of strain 15-3466, which was co-isolated with a FAdV-1 strain.

The analysis of all FAdV-B strains (Figure 2C) indicated, again, the questionable typing result of strain TR22. This strain was isolated in Japan in the 1960s and was serotyped as FAdV-5 [48]. However, our findings confirmed previous results [49], as the strain was not monophyletic with other FAdV-B strains. The serological typing results of this strain remain questionable, and a repeated cross-neutralization assay would be supported.

TR22 was isolated in Japan, and strain 340 in Northern Ireland, but the majority of more recent FAdV-B isolations or detections happened in two geographical regions: Central Europe and West Africa. Since 2007, 31 FAdV-B strains (Table 2) have originated from France (one), Germany (three), Austria (nine), Hungary (sixteen), Ukraine (one) and Russia (one), where the latter strain was isolated in Rostov, about 100 km from the Ukrainian border [10,11,12,13,50,51]. A further two strains (NCBI Nucleotide: MT525091, MW353019), originating from Poland, were excluded from the analysis based on low sequence quality [52]. Moreover, six strains were detected in West Africa: in Cote d’Ivoire (five) and in Ibadan, Southwest Nigeria (one) [53]. Thus, the only geographical exception since 2007 is a single strain from South Korea (HQ697592) [54], although numerous typing results have recently been published worldwide [40,41,42,43,44,45,55,56,57,58,59,60,61,62,63]. Seemingly, species FAdV-B has been limited almost exclusively to Central Europe and West Africa in recent years.

The exact cause of this is not known. It might be hypothesized that FAdV-B strains have adapted evolutionarily to these geographical regions or husbandry technologies, and have persisted there, whereas this species was superseded by other FAdV species in other regions; for example, the trade of hatching eggs is widely practiced in Europe, and enables the spread of a certain serotype due to vertical transmission. Another possibility is the exact contrary of the former: a recent spread of FAdV-B strains in these locations. This might be supported by the emergence of two genotypes within the species: B2, and a novel one, B3. As mentioned before, B2 strains represent 75% of all FAdV-B strains, and a further two strains (isolated in 2012 and 2013) constitute a relatively independent branch, characterized by a long branch length; thus the genotype B3 was established (Figure 2C). This analysis was based on the hexon loop-1 nucleotide sequence, a genomic stretch under the highest evolutionary pressure of the host’s immune system [35]. Other parts of the genome are far from this mutation rate, and intraspecies distances compared on the complete genome (Figure 2A) or the DNA polymerase (Table 3) are minuscule.

The members of almost every FAdV species are associated with specific diseases. Namely, FAdV-A (serotype FAdV-1) with gizzard erosion, FAdV-C (FAdV-4) with hepatitis–hydropericardium syndrome, and FAdV-D (FAdV-2, -11) and FAdV-E (FAdV-8a, -8b) with inclusion body hepatitis [5]. In this respect, species FAdV-B is an exception, since such strains have not been found to be the aetiological agent of a well-circumscribed disease. On the contrary, FAdV-B strains have already been found in connection with tenosynovitis, inclusion body hepatitis, airsacculitis, pericarditis, enteritis, cardiac decompensation or nephrosis in chickens, and have also been isolated from healthy mallards [10,11,12,50,64]. No clear pattern was observed in our study either; FAdV-B was mainly an incidental finding, and lesions were attributed to other viral/bacterial infections diagnosed in the flocks. Strains belonging to this species are most likely facultative pathogens of chickens, and additional immunosuppressive effects are necessary for disease manifestation.

The genome sequences of fourteen FAdV-B strains, representing currently circulating strains in Europe, were determined, and four selected strains were compared using serum cross-neutralization to reference strain 340. Based on previous phylogenetic analyses, it was hypothesized that the divergent FAdV-B strains might represent a novel serotype within the species, but the actual cross-neutralization tests refuted this. This research underlines the importance of traditional neutralization assays, optimally performed as reciprocal testing when diagnostic difficulties are suspected due to prime relationships. Furthermore, we provide a snapshot of the current diversity of the species *Fowl aviadenovirus B*, for which a long-term gap in epidemiological documentation has obviously lead to the widely unrecognized emergence and spread of particular antigenic variants.

## Figures and Tables

**Table 3:** Pairwise sequence identity values of *Fowl aviadenovirus B* strains.

Analyzed Stretch	Sequence Identity of the Newly Sequenced Strains to the Two Strains:	
	340	40440
Complete genome (NA)	98.50–98.54%	97.97–97.98%
DNA polymerase (AA)	99.46–99.54%	99.07–99.15%
Penton base (AA)	99.63%	100.00%
Hexon complete (AA)	97.27%	100.00%
Hexon loop-1 (AA)	89.94%	100.00%
Fiber (AA)	92.93%	96.56%
Fiber knob (AA)	89.80%	100.00%
Concatenate (AA) *	95.40%	100.00%

* Concatenate of antigenic determinants: penton base, hexon loop-1, hexon loop-2 and fiber knob amino acid sequences. Abbreviations: AA—amino acid; NA—nucleic acid.

**Table 4:** Cross-neutralization testing between selected FAdV-B field isolates and the historical reference strain 340. The extent of neutralization is expressed as the reciprocal titer of antisera, standardized to 20 serum units against 100 TCID_50_ of the homologous virus. Values highlighted in grey represent the homologous neutralization and the reference for all other reactions in the respective vertical column.

Virus (100 TCID_50_)	Antiserum (Reciprocal Titer)				
	40440	15-6541	15-4225	18-6238	340
**40440**	640	640	1280	640	-
**15-6541**	320	640	1280	640	-
**15-4225**	640	640	1280	640	-
**18-6238**	320	640	1280	640	-
**340**	320	320	320	160	320

-, No neutralization was recorded.

**Table 5:** Amino acid substitutions of the major capsid proteins.

Protein	340 Versus 40440					340 Versus FAdV-8a				
	Number of Diff.	Charge-Altering Substitutions				Number of Diff.	Charge-Altering Substitutions			
		Polar-to-Charged ^a^	Nonpolar-to-Charged ^b^	Charge Reversal ^c^			Polar-to-Charged ^a^	Nonpolar-to-Charged ^b^	Charge Reversal ^c^	Insertion/Deletion ^d^
**Hexon**	26	5	1	0		126	9	9	2	0
**Fiber knob ^e^**	24	2	2	0		113	14	8	1	1
**Penton base ^f^**	1	1	0	0		63	10	2	0	1
**Sum**	51	8	3	0		302	33	19	3	2

Charge-altering substitutions: ^a^ Polar-to-charged—the substitution of a polar, uncharged amino acid by a charged one or vice versa. ^b^ Nonpolar-to-charged—the substitution of a nonpolar amino acid by a charged one, or vice versa. ^c^ Charge reversal—the substitution of a positively charged amino acid by a negatively charged one or vice versa. ^d^ Insertion/deletion—number of charged amino acids in insertions and deletions. ^e^ Amino acid stretches for strain 340: 349–553, 40440: 349–554, FAdV-8a: 314–524. ^f^ Amino acid stretches for strain 340: 76–542, 40440: 77–543, FAdV-8a: 81–552.

## Data Availability

The complete genome sequences were deposited to the GenBank, under accession numbers OK283042-OK283055, whereas sequence reads were archived under the NCBI BioProject accession number PRJNA781911.
